# Narratives in Conflict and Practices of Face-to-Face and Online Intergroup Communication

**DOI:** 10.3390/bs16020231

**Published:** 2026-02-05

**Authors:** Yiftach Ron

**Affiliations:** 1 School of Creative Arts Therapies, Faculty of Humanities and Social Sciences, Kibbutzim College, Tel Aviv 6250769, Israel; yiftach.ron@smkb.ac.il; 2 The Swiss Center for Conflict Research and the Department of Communication, Faculty of Social Sciences, The Hebrew University of Jerusalem, Jerusalem 9190501, Israel

**Keywords:** group behavior, intergroup communication, ethno-political conflict, narrative, online dialogue

## Abstract

Intergroup communication (IC) serves as a critical arena in which narratives, worldviews, and group behaviors are expressed, confronted, and translated into concrete communicative practices. Within this unique space of interaction, divergent narratives may remain rigid and unchanging, manifesting as parallel monologues that coexist without genuine engagement. Yet, under certain conditions, such communication can also open the door to dynamic processes of mutual challenge, development, and transformation. This narrative literature review aims to strengthen the growing connection between the scholarship on narratives in societies embroiled in intractable conflict and the well-established research tradition on intergroup contact. Specifically, it seeks to enhance our understanding of the interplay between narratives, behaviors, and communication practices in both face-to-face (FTF) and online contexts of IC. While the discussion includes broader global perspectives, the primary case study centers on the ongoing conflict and communicative interactions between Israeli Jews and Palestinians.

## 1. Introduction

Research on narratives, identities, and group behaviors in contexts of intractable ethno-political conflict highlights the central role of collective narratives in perpetuating conflict. These narratives serve to consolidate exclusive and polarized national identities, contribute to the delegitimization of the opposing group, and shape perceptions of the conflict as a zero-sum game (Bliuc & Chidley, 2022; Bekerman & Zembylas, 2009, 2011; Ehrmann & Millar, 2021; Gamaghelyan & Rumyantsev, 2021; Hammack, 2011; Jelić et al., 2021; Ventsel et al., 2019; Whittle, 2023). A complementary body of literature explores psychological intergroup interventions aimed at improving intergroup relations and mitigating intergroup conflicts (Bar-Tal & Hameiri, 2020; Čehajić-Clancy & Halperin, 2024; Halperin et al., 2024). Within this framework, extensive research examines the potential of face-to-face (FTF) encounters and online intergroup communication (IC) processes to reduce prejudice, intergroup anxiety, and hostility, while fostering peacebuilding efforts (Burrows et al., 2022; Mor et al., 2016; Nolte-Laird, 2022; Pettigrew & Tropp, 2011; Ron et al., 2020; Stephan, 2014; Tropp et al., 2022; Van Assche et al., 2023).

This *narrative review* seeks to explore the interplay between collective narratives, group behaviors, and communication practices in the context of intractable conflict. It aims to shed light on the unique role IC may play in shaping the ethos, narratives, and ideologies that characterize ethno-political conflicts, as well as its potential contribution to the fields of conflict resolution and peacemaking.

It should be noted that the review incorporates several terms, sometimes with highly similar semantics, depending on how they are presented in the studies under discussion. Thus, for example, the term ‘narrative engagement’ (Busselle & Bilandzic, 2009; Hammack, 2009) refers to the process by which individuals are cognitively and emotionally influenced and become immersed in the narratives they consume. The term ‘ethos of conflict’ (Bar-Tal, 2013; Ben David et al., 2017; Maoz & Ron, 2016; Ron & Maoz, 2013a; Zigenlaub & Sagy, 2020) refers to the comprehensive, deeply embedded shared beliefs, values, and emotions held by a society involved in a prolonged, intractable conflict. The term ‘master narrative’ (Hammack, 2009, 2011) refers to a cultural script or a dominant discourse that proliferates in a particular society. These terms are introduced alongside more common and familiar terms, such as ‘collective narratives’ and ‘collective memory’. Section 2 of this review demonstrates how these concepts represent key constructs in the formation of narratives within intergroup contexts, and particularly in situations of intractable conflict. In Section 3, these constructs are linked to different IC models and frameworks, illuminating the contribution they seek to offer in settings of intergroup conflict.

### Search and Selection Method

As noted above, this review is defined as a *narrative literature review*. Whereas highly structured methods, such as *systematic review* or *meta-analysis*, are bound by narrowly defined parameters and precise inclusion and exclusion rules, a narrative review has more flexibility, allowing the author to utilize their expertise for the selection, interpretation, and synthesis of sources (Bourhis, 2017). Due to its ability to integrate insights from diverse disciplines, and enable a deeper understanding of complex issues, this method is particularly valuable in fields with a high literature diversity and is considered to have unique advantages in the study of interdisciplinary and emerging topics (Ahmad, 2025; Greenhalgh et al., 2018).

A narrative review does not strictly require the reporting of methodological components such as search terms, specific databases, or formal inclusion and exclusion criteria (Bourhis, 2017; Jahan et al., 2016). Nevertheless, the sourcing for this review was conducted in a systematic manner. The article collection process utilized the *snowballing method*, commencing with a limited set of 21 core articles selected based on the author’s expertise, and expanding outward from there. This involved both *backward snowballing*, where reference lists were systematically reviewed to find earlier relevant works, and *forward snowballing*, using citation tracking databases (Google Scholar and Scopus) to identify more recent studies. The selection process focused on two criteria: relevance to the review topic and recency. Relevance was assessed based on the author’s professional judgment, while recency was predefined as the past seven years, though older works were included for contextual and other purposes. The screening process involved thousands of sources, approximately 180 of which were ultimately included in the review. Finally, the articles were summarized to facilitate an integrative synthesis aligned with the review’s objectives.

The selection of the case study presented in this review—the ongoing conflict and communicative interactions between Israeli Jews and Palestinians—is grounded in three central considerations. First, the author possesses distinct expertise in this field, which facilitates the utilization of expert judgment. This is a core advantage of the narrative review approach, as it enables in-depth interpretation and synthesis across various approaches and disciplines (Ahmad, 2025). Second, several prominent works addressing the interface between conflict, narratives, and IC focus specifically on the Israeli–Palestinian conflict (see Section 5 below). Lastly, the salience, strong resonance and heightened urgency surrounding the conflict and the relationship between Israelis and Palestinians—particularly in the years since October 2023—constitute a further consideration in the selection of the case study.

## 2. Narratives and Their Role in Intergroup Conflicts

### 2.1. Narratives as Concept

Narratives have become a central concept in the study of social and cultural phenomena. Bruner (2008) and Myers (2021) argue for the primacy of narrative as a fundamental organizing principle of cultural life. By providing meaning, narratives form the salient content of the mind and reveal links to a community of shared stories and practices (Bruner, 1990; Myers, 2021). Through the process of narrative engagement (Busselle & Bilandzic, 2009; Hammack, 2009; Sukalla et al., 2016), individuals construct personal life stories that, while distinct from collective narratives and the broader structures of social identity, are nonetheless grounded in and shaped by them, simultaneously serving to carry them forward (Bliuc & Chidley, 2022; Bamberg, 2011; Hammack, 2009; Kawai et al., 2022).

At the group level, narratives function as a tool of mobilization, reinforcing social identity by converging around shared historical memories and ‘meta-stories’ that incorporate moral codes and values (Bliuc & Chidley, 2022), and constructing a meaningful and coherent sense of the past and the contemporary reality (Bhat et al., 2023; Grever & van der Vlies, 2017; Haraldsson & McLean, 2022; Liu & Hilton, 2005; Obradović & Bowe, 2021). They contribute to the formation of social consensus, strengthen feelings of solidarity and belonging to political, national, or religious communities (Bekerman & Zembylas, 2009), and provide justification for their existence and modes of operation, both internally and externally (Bliuc & Chidley, 2022; Gamaghelyan & Rumyantsev, 2021).

Collective narratives provide social representations of collective history and serve as expressions of collective memory, contributing to the group’s social distinctiveness (Hammack, 2011; Nicolson & Korkut, 2021). As such, they constitute a central component in the construction of group identity and in shaping attitudes toward other groups (Bliuc & Chidley, 2022; Figueiredo et al., 2017; Liu & Hilton, 2005; Obradović & Bowe, 2021; Uluğ, 2023). Narratives structure historical memory by assigning prominence to certain events over others (Bhat et al., 2023; Grever & van der Vlies, 2017), thereby defining the terms through which reality is described. They also reflect the prevailing ideology, norms, and values of the society in which they emerge (Bekerman & Zembylas, 2009, 2011), while simultaneously revealing the deep fears, perceived threats, and historical traumas that fuel the conflicts in which groups are engaged (Auchter, 2018; Li et al., 2022; McLamore et al., 2019; Ross, 2002).

At the individual level, narrative functions as a structural mechanism through which people make sense of their ideological affiliations and personal experiences (Alber, 2017; Bamberg, 2011; McLean et al., 2017, 2020). This process occurs, among other means, through the construction of temporal and spatial frameworks, as well as contexts of causality and continuity (Haraldsson & McLean, 2022; McAdams, 2021; McAdams et al., 2006). Narratives serve as interpretive anchors for experience and support the ongoing process of identity formation (Bamberg et al., 2011; De Fina & Georgakopoulou, 2011; Hammack, 2011; McLean et al., 2017, 2020; Park & Moon, 2022). They foster a sense of safety and meaning, enhance coherence (Bekerman & Zembylas, 2009; Duranti, 2006; Haraldsson & McLean, 2022), and contribute to the development of positive self-esteem (Bamberg, 2011; Bar-On, 2006; Hilman et al., 2023).

Personal narratives reveal how people position themselves within systems of power relations in society, as well as how they internalize prevailing social discourses at a given moment (Bamberg et al., 2011; De Fina et al., 2006; Hammack, 2011; Hammack & Pilecki, 2012; McLean et al., 2020). In doing so, they shed light on the processes through which thoughts, perceptions, and emotions are structured, offering insights into how individuals and groups make sense of their social worlds and the conflicts in which they are involved (Hammack, 2009; Kvernbekk & Bøe-Hansen, 2017; Ron & Maoz, 2013a).

### 2.2. Narratives in Intergroup Conflict

In contexts of intergroup conflict, narratives play a crucial role in reinforcing the legitimacy of the group and its actions (Bekerman & Zembylas, 2009; Bliuc & Chidley, 2022; Gamaghelyan & Rumyantsev, 2021), in shaping group identity, and in enhancing the sense of meaning, order, security, and social solidarity (Alayan & Riley, 2024; Buheji & Hamza, 2024; Canetti et al., 2017; Hammack & Pilecki, 2012; Klar & Baram, 2014; Uluğ & Cohrs, 2017). Simultaneously, narratives help individuals and communities cope with trauma and emotional pain (Jirek, 2016; Kearney, 2007; Witteborn, 2007), offering a response to the anxieties, perceived threats, and feelings of insecurity that accompany situations of intergroup tension and violence (Hammack, 2009, 2011; Jelić et al., 2021).

Narratives also play a central role in sustaining and even exacerbating intergroup conflict (Bar-Tal, 2020; Bekerman & Maoz, 2005; Golynchik, 2020; Rotberg, 2006; Uluğ et al., 2020). Group narratives, inherently limited in both their capacity to represent others and in the quality of those representations, tend to become particularly exclusive and selective in conflict settings (Bar-On & Adwan, 2006; Ehrmann & Millar, 2021; Tyushka, 2021; Ushomirsky et al., 2023). They offer partial and narrow interpretations of the conflict that serve to justify the group’s actions and portray it as a victim, while simultaneously excluding, morally denouncing, and even delegitimizing the other and their narrative (Bekerman & Zembylas, 2009, 2011; Bliuc & Chidley, 2022; Cole, 2007; Hameiri & Nadler, 2017; Hameiri et al., 2024; Jeong et al., 2023; Tyushka, 2021; Uluğ et al., 2020).

Especially in situations of intractable conflict (Bar-Tal, 2013, 2020; Golynchik, 2020; Harel et al., 2024; Zartman, 2019), the opposing narratives of both sides are marked by the absolute justification and idealization of the in-group, alongside the cultivation of a collective identity rooted in victimhood and the simultaneous dehumanization and demonization of the out-group (Bar-Tal, 2020; Bliuc & Chidley, 2022; Maoz & McCauley, 2008; Zembylas & Bekerman, 2008). These competing group narratives impose a heavy emotional burden on the conflict, characterized by fear, contempt, blame, and resentment (Bar-Tal & Salomon, 2006; Bliuc & Chidley, 2022; Jelić et al., 2021), and are accompanied by a socio-psychological repertoire of attitudes, goals, and beliefs regarding the causes and trajectory of the conflict, the nature of the adversary, and the vision of a desirable resolution (Bar-Tal, 2020; Ehrmann & Millar, 2021; Bliuc & Chidley, 2022;). In doing so, conflict-sustaining group narratives contribute to the entrenchment and perpetuation of a broader ethos and culture of conflict (Adwan, 2024; Bar-Tal, 2020; Golynchik, 2020).

One of the most prominent test cases illustrating the role narratives play in intergroup relations is the Israeli–Palestinian conflict. In a series of studies focusing on Israeli and Palestinian youth, Hammack (2009, 2011) examines the role and influence of master narratives, which he defines as cultural scripts or dominant discourses that proliferate within a society (Hammack, 2009). He emphasizes the significant role these master narratives play in sustaining the Israeli–Palestinian conflict and reproducing it across generations. According to Hammack, the collective narratives embedded in the discursive field of the conflict reinforce the polarized national identities of both groups, as well as the belief systems and emotional frameworks that have fueled the conflict over time (Hammack, 2009, 2011).

Other scholars draw similar conclusions. Bar-On and Adwan (2006) and Adwan (2024) describe how each side’s narrative aligns with what they term the logic of conflict and contention, relying on discursive codes of intergroup exclusion. Building on this, Bekerman and Zembylas highlight the pervasive influence of collective narratives and national discourse even among those engaged in educational efforts aimed at coexistence (Bekerman & Zembylas, 2009, 2011). Rotberg (2006) further illustrates how historical consciousness and competing historical narratives continue to shape contemporary emotions and positions among both Israelis and Palestinians. In a similar vein, other scholars describe the role of collective traumas, memory, and narrative in structuring mutually exclusive national identities and framing the conflict as a zero-sum game (Alayan & Riley, 2024; Bar-Tal, 2020; Buheji & Hamza, 2024; Dajani Daoudi & Barakat, 2013; Klar & Baram, 2014).

Alongside the role of collective memory and master narratives in sustaining intergroup conflicts, several scholars point to the phenomenon of competing narratives held by different ideological groups and sectors within societies involved in intractable conflict (Bar-Tal et al., 2014; Oren et al., 2015). While external threats may lead to greater intragroup cohesion in certain cases, a growing body of research challenges the traditional assumption regarding the unifying effect of such threats, and suggests that intragroup conflict between competing narratives can also emerge or escalate, potentially leading to phenomena such as intragroup affective polarization within societies embedded in intractable intergroup conflict (Harel et al., 2020, 2024).

Given the central role narratives often play in escalating and perpetuating ethno-political conflicts, it is essential to examine how IC processes can challenge conflict-supporting narratives and behaviors and contribute to reconciliation and peace-building. The following section examines how various IC models—most notably, but not exclusively, the narrative model—engage with the foundational constructs of the ethos and culture of conflict, including personal and collective narratives, collective traumas and memories, and the master narratives of societies embroiled in intractable conflict.

## 3. Intergroup Communication in Intractable Conflicts

Intergroup communication (IC), also referred to as intergroup dialogue or intergroup contact, is widely employed as a strategy for improving relations between groups. Zúñiga (2003) and Zúñiga et al. (2014), who used the term intergroup dialogue, defines it as “a face-to-face facilitated conversation between members of two or more social identity groups that strives to create new levels of understanding, relating, and action” (Zúñiga, 2003, p. 9). Among the various interventions designed to reduce intergroup bias, IC has been the most widely applied and the most extensively studied (Al Ramiah & Hewstone, 2013; Pettigrew & Tropp, 2011; Ron et al., 2017, 2020; Tropp et al., 2022).

The starting point for most theoretical discussions of IC is Allport’s (1954) contact hypothesis. This hypothesis posits that intergroup contact can reduce stereotypes when four key conditions are met: (1) equal status among the groups within the contact setting, (2) shared goals and cooperation in achieving them, (3) opportunities for personal acquaintance through sustained and meaningful interaction, and (4) social and institutional support for the contact. Subsequent researchers have proposed additional conditions for successful IC, including the use of a common language, voluntary participation, interactions that are perceived as pleasant and mutually beneficial, favorable economic circumstances, and the absence of strongly negative attitudes toward the out-group (Dixon et al., 2005, 2007; McKeown & Dixon, 2017).

Most empirical studies examining the impact of IC on reducing prejudice have reported positive outcomes when it occurs under the conditions outlined in the original contact hypothesis, even when not all conditions are fully met (Al Ramiah & Hewstone, 2013; Burrows et al., 2022; Ellis et al., 2016; Pettigrew & Tropp, 2006, 2011; Tropp et al., 2022; Van Assche et al., 2023). At the same time, scholars have criticized the hypothesis for its limitations, particularly regarding its narrow focus on the individual level of the IC experience, its difficulty in sustaining positive effects during periods of escalating intergroup conflict, and its limited capacity to address issues of interethnic relations and asymmetric power dynamics (Bekerman, 2009; Dixon et al., 2005, 2007; Kende et al., 2017; Maoz, 2010, 2011; McKeown & Dixon, 2017; Reimer & Sengupta, 2023; Saguy & Dovidio, 2013; Suleiman, 2004).

In light of the limitations of the contact hypothesis, alternative approaches to IC have been developed. Maoz (2011) identifies four distinct IC models (see Table 1 at the end of the section), each highlighting different aspects of intergroup interaction:

1. The coexistence model emphasizes interpersonal communication, fostering understanding and tolerance, and reducing stereotypes, with particular focus on similarities and shared aspects (Allport, 1954; Dixon et al., 2005; McKeown & Dixon, 2017). In terms of narratives within intergroup conflict, this model primarily addresses personal narratives, images of the ‘other’, and prejudice through interpersonal acquaintance and an emphasis on commonalities.
2. Building on this interpersonal focus, the joint project model assumes that cooperation on a shared task directed toward a mutually relevant goal can strengthen relations between groups and promote the formation of a superordinate identity (Sherif, 1966; Sherif et al., 1961; Smith & Haslam, 2017; Swaab et al., 2021). Indirectly and implicitly, this IC model can be viewed as an attempt to influence narratives of the self and the ‘other’ by fostering a superordinate identity.
3. In contrast, the confrontational model shifts attention to the conflict itself, underscoring the importance of acknowledging asymmetric power relations and emphasizing group and national identities, in line with the social identity theory tradition (Hogg et al., 2017; Tajfel & Turner, 1979, 1986). Unlike the coexistence and joint project models, the confrontational model engages with collective constructs such as the ethos of conflict, collective identities and memories, and the master narratives of societies embroiled in intractable conflict.
4. The narrative model, extending this acknowledgement-based approach, builds on storytelling (Bar-On, 2010; Husnu et al., 2018) and stresses the importance of exposure, recognition, and legitimization of the other’s narrative (Adwan, 2024; Bar-On, 2010; Castro et al., 2023; Salomon, 2004; Zigenlaub & Sagy, 2020). As such, the narrative model addresses both individual and collective manifestations of the role that narratives play in intergroup conflict settings.

The narrative model is closely associated with Salomon’s (2004) and Kupermintz and Salomon (2006) theoretical approach to intergroup reconciliation and, even more strongly, with the theory and practice developed by Bar-On (2006, 2010) and Ron and Maoz (2013a). Salomon argued that the collective narratives of groups in conflict and their implicit delegitimization of the out-group’s narrative should be the primary target for change in efforts to promote reconciliation. To this end, he proposed an educational process centered on exposing, recognizing, and legitimizing the narrative of the other (Kupermintz & Salomon, 2006; Ron & Maoz, 2013a; Salomon, 2004).

Bar-On’s approach to encounters and communication between conflicting narratives was embodied in the development of the TRT (To Reflect and Trust) groups (Bar-On, 2006, 2010). Initially implemented in encounters between descendants of Jewish Holocaust survivors and descendants of Nazi perpetrators and later adapted to IC processes in various conflict zones, the TRT groups emphasized sharing, listening, and reflecting on personal stories, with the aim of enabling mutual coping with traumatic pasts as well as contemporary realities (Bar-On, 2010; Mana & Srour, 2020).

Ron and Maoz (2013a), Maoz and Ron (2016) and Ron (2022) highlight the potential of confronting the other’s narrative within IC processes to foster a new and more critical awareness among participants, constituting what they term ‘dangerous stories’ (Ron & Maoz, 2013a). These are new and subversive practices of remembering the past and interpreting contemporary reality (Bekerman & Zembylas, 2011; Ostovich, 2002, 2005) that challenge taken-for-granted and fixed narratives, as well as tribal group identities, thereby opening possibilities for alternative affective and ethical relations with others (Ron & Maoz, 2013a). These findings align with those of Murrar and Brauer (2018, 2019) concerning the potential role of narratives as a useful tool for fostering more positive attitudes toward out-group members and improving intergroup relations. Furthermore, they are consistent with the findings of Bilali and other scholars regarding the ability of narrative interventions—including communication of social norms, role modelling of behaviors, perspective-taking, awareness-raising, and imagined contact—to contribute to the reduction in prejudice and violence, and to promote reconciliation in contexts of intergroup conflict (Bilali, 2024; Murrar & Brauer, 2018).

Thus, the narrative approach recognizes the central role of collective and personal narratives in sustaining intractable conflicts, and therefore emphasizes the need to address these deep-rooted narratives while exposing participants to the narrative of the other through IC processes (Kim & Lim, 2021; Murrar & Brauer, 2019; Ron & Maoz, 2013a; Scham et al., 2013; Zigenlaub & Sagy, 2020).

## 4. Online Intergroup Communication in Intractable Conflicts

Over the past 25 years, online communication has become a significant platform for enabling new forms of social contact and interaction (Mor et al., 2016; Hasler et al., 2023; Ron et al., 2020). The rapid expansion of social networks has facilitated a shift toward novel modes of communication and engagement (Bennett & Segerberg, 2012; Castells, 2013; Papacharissi, 2002; Ron et al., 2020; Vaccari & Valeriani, 2021), contributing to the emergence of new centers of power and influence. These developments have also allowed for the expression and dissemination of alternative narratives surrounding social, political, and ethno-political realities (Blagojević & Šćekić, 2022; Castells, 2013; Cortés-Ramos et al., 2021; Curran, 2002; Lorenz-Spreen et al., 2023; Ron et al., 2020).

The wave of protests across the Arab world that began in January 2011 drew increased academic attention to the role of social media in mobilizing solidarity and civil action (Aouragh, 2012; Blagojević & Šćekić, 2022; Wilson & Dunn, 2011). Within this broader context, scholars have also begun to explore the potential of social media to build societal bridges, serving as a new space in which individuals from opposing groups, holding conflicting narratives about the intergroup situation, can come together and engage in a communication process that fosters mutual challenge, narrative development, and potential transformation of worldviews related to the conflict (Ellis & Maoz, 2007; Hasler & Amichai-Hamburger, 2013; Hasler et al., 2023; Mitchell, 2009; Nolte-Laird, 2022; Ron et al., 2020; Walther et al., 2015).

While IC processes have traditionally taken place primarily in FTF settings, recent years have seen growing scholarly attention to interventions based on online IC (Amichai-Hamburger et al., 2015; Hasler & Amichai-Hamburger, 2013; Nagar et al., 2021; Nolte-Laird, 2022; Schwab et al., 2018; Segal & Keduri, 2022). In the literature, these digitally mediated forms of intergroup engagement are commonly referred to as computer-mediated communication (CMC), online intergroup dialogue (Ellis & Maoz, 2007; Mor et al., 2016; Nolte-Laird, 2022; Ron et al., 2020; Selvanathan et al., 2019) or virtual contact (Hebel-Sela et al., 2025). These forms of communication become particularly relevant as they help overcome the limitations and difficulties associated with facilitating face-to-face encounters—including geographical distances, physical barriers, movement restrictions, and entrenched patterns of segregation and intergroup anxiety—that often separate groups in conflict, especially when outbreaks of violence render physical meetings impossible (Hasler & Amichai-Hamburger, 2013; Hebel-Sela et al., 2025; Mor et al., 2016).

When examining studies on online IC, a distinction should be drawn between the established field of IC on social media, the relatively limited body of research on structured interventions via video conferencing or text-based exchanges, and the more recently emerging domain of communication facilitated by technological advancements such as Virtual Reality (VR) and Artificial Intelligence (AI). In the context of social media communication, as well as through structured interventions based on video conferencing or text exchanges, several studies have examined IC processes through the lens of intergroup contact theory (Allport, 1954; McKeown & Dixon, 2017), identifying communication patterns that also characterize FTF encounters (Amichai-Hamburger et al., 2015; Hasler et al., 2023; Imperato et al., 2021, 2024; Mor et al., 2016; Ron et al., 2020; Selvanathan et al., 2019). Drawing on Maoz’s (2011) typology of IC models, various studies highlight the effectiveness of social media and other forms of intergroup contact as platforms for facilitating IC processes aligned with the coexistence model of intergroup contact. This model, grounded in Allport’s (1954) contact hypothesis, emphasizes interpersonal engagement, the promotion of mutual understanding and tolerance, and a focus on intergroup commonalities (Imperato et al., 2021, 2024; Maoz, 2011; Mor et al., 2016; Ron et al., 2020; Schwab et al., 2018). Evidence for the effectiveness of online communication oriented toward the coexistence model can be found in studies examining online interventions, particularly collaborative learning and joint projects among teachers, students, and faculty, spanning several months to an academic year. These studies found a reduction in out-group prejudice among the participants (Hoter et al., 2012; Nagar et al., 2021; Walther et al., 2015). In the context of IC on social media, research examining participants in unstructured intergroup interactions on Facebook pages designed for non-violent intergroup communication found a correlation between participation in such virtual interactions and lower levels of prejudice toward the respective out-group (Schwab et al., 2018).

However, social media interactions oriented toward the confrontational model of intergroup contact, which focuses on group identities and asymmetric power relations in the context of ongoing political and social conflicts (Maoz, 2011; Ron et al., 2010), as well as those grounded in the narrative model, in which participants from both groups engage in story-telling by sharing personal and collective narratives, experiences, and suffering related to the conflict (Bar-On, 2008; Zigenlaub & Sagy, 2020), have not been found effective in reducing intergroup hostility and were often accompanied by a backlash effect (Hasler et al., 2023; Ron et al., 2020). Two studies based on content analysis of posts and comments exchanged between Israeli Jews and Palestinians on Facebook pages aimed at intergroup dialogue and collective action—one examining posts over a month-long period of escalated conflict (Mor et al., 2016) and the other over ten months characterized by a relative lull in conflict-related violence (Ron et al., 2020)—revealed how exposure to the other side’s narrative and calls for political action often led to negative, defensive responses and mutual recriminations.

Mor et al. (2016) suggest that the limited effectiveness of social media for supporting more complex models of intergroup dialogue, such as the confrontational and narrative approaches, may stem from the discontinuous nature of interactions on social networks. These platforms often lack the processual depth and continuity necessary for dynamic engagement capable of challenging conflict-supporting beliefs, worldviews, and narratives (Maoz & Ron, 2016; Mor et al., 2016; Nolte-Laird, 2022). Given these limitations, which may even result in backlash effects in intergroup dynamics (Ron et al., 2020), some scholars recommend adapting the goals and design of IC interventions conducted on social media to the constraints inherent to these platforms (Hasler et al., 2023; Imperato et al., 2021; Mor et al., 2016). Another possible direction is to explore the potential of technological developments, such as VR and AI, to promote IC processes and help improve intergroup relations. VR uses computer technology to create a simulated environment (Kabiljo, 2019). Several studies examining the use of VR to simulate intergroup encounters point to the potential of this tool as a means of eliciting empathy and creating a sense of closer, more harmonious interaction through computed mimicry of the virtual interlocutor. These effects were observed even among participants with negative baseline attitudes toward the out-group (Hasler et al., 2014). Regarding the use of AI, recent studies point to the ability of AI-driven contact interventions to evoke parasocial closeness (one-sided emotional connection with media figures—in this case, artificial agents representing the out-group) and thus help reduce prejudice and intergroup anxiety while promoting out-group perspective-taking and a greater openness to attitude change (Manfredi et al., 2025). Such use of AI could serve as a preliminary step that softens emotional resistance towards more complex IC interventions. It should be noted that this research direction is still in its infancy (Manfredi et al., 2025).

## 5. Intergroup Communication and the Israeli–Palestinian Conflict

The protracted dispute between Israelis and Palestinians falls into the category of intractable conflicts, which are characterized by totality, violence, and a widespread perception of being irresolvable. Such conflicts dominate each side’s agenda and shape the logic and perspectives of the societies involved (Bar-Tal, 2013; Bar-Tal et al., 2009; Dajani Daoudi & Barakat, 2013; Golynchik, 2020; Harel et al., 2024, McLamore et al., 2019; Uluğ et al., 2020; Zartman, 2019). The Israeli–Palestinian conflict is further marked by asymmetrical power relations, specifically, the political and military domination of Palestinians by Israel (Bar-On & Adwan, 2006; Buheji & Hamza, 2024; Kriesberg, 2009; Ron & Maoz, 2013b). Although the Palestinian Territories include semi-autonomous regions designated under the 1993 Oslo Accords, and despite the Gaza Strip having enjoyed even greater autonomy from 2005 until the outbreak of the Gaza War in 2023, Israel continues to exercise effective control over the Territories and regularly conducts military operations there (Bar-On & Adwan, 2006; Hammack, 2010; Hughes, 2020).

As for relations between Jews and Palestinians within the State of Israel, Maoz (2004) identifies two main characteristics of the sociopolitical context that are particularly relevant to IC processes between the two groups: (1) a coexistence marked by aggression alongside cooperation, and (2) inequality, whereby Jews enjoy greater access to resources and stronger influence over the culture, religion, and language of the State.

The psychological and behavioral repertoire associated with intergroup conflicts plays a significant role in shaping IC processes. Several studies (Halperin, 2011; Halperin & Bar-Tal, 2011; Hameiri et al., 2017; Leshem & Halperin, 2022) have documented the psychological dynamics of the Israeli–Palestinian conflict, in which each side blames the other for initiating and perpetuating the conflict and for its violent nature, while simultaneously perceiving itself as the victim and as genuinely seeking peace. Sheehi and Sheehi (2016) term these defensive dynamics ‘enactments of otherness’, arguing that their outcome is the preclusion of a genuinely reciprocal inter-subjective space of relatedness. These perceptions and attitudes are precisely what IC interventions between Jews and Palestinians aim to address.

While the psychological mechanisms and dynamics described above characterize individuals and societies in other areas of intractable conflict as well, the relationships and IC processes between Israeli Jews and Palestinians also possess unique characteristics that may not necessarily apply to other regions or contexts of ethno-political conflict. Some of these pertain to the relations between Jews and Palestinians within the State of Israel. Over the past four decades, Jewish–Palestinian encounters have engaged with issues related not only to the broader Israeli–Palestinian conflict but also to the relationship between the Jewish majority and the Arab Palestinian minority within Israel. Specifically, these encounters have focused on attitudes and beliefs concerning the causes and trajectory of the Israeli–Palestinian conflict, as well as visions of its resolution (Maoz & Ron, 2016; Mor et al., 2016; Ron, 2022; Ron & Maoz, 2013b). They have also addressed majority–minority relations within Israel, including issues of discrimination and civic equality, as well as questions of belonging and loyalty (Bekerman, 2009; Ben David et al., 2017; Kahanoff, 2016; Ron et al., 2010; Zembylas & Bekerman, 2008).

Several stages in the evolution of structured IC between Israeli Jews and Palestinians have been documented in the research literature (Abu-Nimer, 2004; Maoz & Ron, 2016; Ron & Maoz, 2013a). In the early phase, up until the early 1980s, Jewish–Palestinian FTF encounters primarily focused on cultural aspects, aiming to expose participants to one another’s culture through shared experiences involving food, dance, and folklore. Then, beginning in the early 1980s, some programs began to adopt a prejudice-reduction approach, imported from the United States (Maoz, 2011; McKeown & Dixon, 2017; Tropp et al., 2022), which concentrated on addressing stereotypes and mutual perceptions. Toward the late 1980s, a more confrontational approach started to emerge in several programs (Maoz, 2011; Ron & Maoz, 2013a). This phase shifted the focus toward the conflict itself, specifically, the clash between Jewish and Palestinian national identities and the broader political context, with the aim of helping participants understand the conflict’s impact on their lives (Abu-Nimer, 2004; Halabi & Sonnenschein, 2004; Maoz, 2011; Ron, 2022).

However, over the past two decades, IC processes aimed at Israeli–Palestinian reconciliation have increasingly adopted a narrative approach (Bar-On, 2006, 2010; Maoz, 2011). This approach acknowledges the central role of both collective and personal narratives in sustaining the conflict and thus emphasizes the need to engage with these deep-rooted narratives and expose participants to the perspective of the other through IC processes (Bar-On, 2010; Mana & Srour, 2020; Maoz & Ron, 2016; Ron & Maoz, 2013a). Empirical studies focusing on the narrative approach suggest that exposure to the other’s narrative in the context of the Israeli–Palestinian conflict, particularly through FTF encounters, can serve as a transformative experience, with the potential to alter deeply entrenched beliefs related to the ethos of conflict (Ben David et al., 2017; Maoz & Ron, 2016; Ron & Maoz, 2013a; Zigenlaub & Sagy, 2020). These findings are not unique to IC processes between Israelis and Palestinians. Similar evidence regarding the role and transformative potential of narratives in the context of intergroup encounters has also been documented in other settings of ethno-political conflict (Ivanović et al., 2024; Jelić et al., 2021).

In addition to these dimensions, various studies highlight the ideological implications of IC processes, both in the context of relations between the Jewish majority and the Arab-Palestinian minority within Israel (Ron et al., 2010, 2020; Sapir & Alimi, 2023), and in terms of their potential to influence attitudes regarding the nature of the Israeli–Palestinian conflict and its possible resolutions (Ellis, 2020; Khalaf & Bekerman, 2024; Ron & Maoz, 2013b; Sheehi & Sheehi, 2016; Thiessen & Darweish, 2018). Although these contexts possess characteristics that are unique to the Israeli–Palestinian conflict, the findings regarding the ideological implications of IC processes add to the broader picture emerging from previous research, which points to the capacity of IC to shift perspectives, challenge deeply held beliefs, and generate ideological and political change (Çakal et al., 2021; Dixon et al., 2005, 2007; Hässler et al., 2021).

## 6. Discussion

This narrative review seeks to strengthen the important link between two evolving areas of scholarship: the growing body of research on narratives in intergroup conflict and the well-established tradition of studying contact and communication processes aimed at improving relations between opposing groups.

Research on narratives and group identities in the context of intractable ethno-political conflicts highlights the significant role collective narratives play in sustaining and even escalating the conflict (Bekerman & Maoz, 2005; Bekerman & Zembylas, 2009, 2011; Golynchik, 2020; Sheehi & Sheehi, 2016; Uluğ et al., 2020). These narratives often serve to reinforce exclusive and polarized collective identities that delegitimize the other side (Bliuc & Chidley, 2022; Ehrmann & Millar, 2021; Hameiri & Nadler, 2017; Hameiri et al., 2024; Hammack, 2011; Tyushka, 2021; Ushomirsky et al., 2023), shape conflict-related emotions and beliefs (Adwan, 2024; Alayan & Riley, 2024; Jeong et al., 2023; Salomon, 2004; Witteborn, 2007), and frame the conflict as a zero-sum game (Bar-Tal, 2020; Bar-Tal & Salomon, 2006; Klar & Baram, 2014).

Despite widespread recognition of the negative role collective narratives play in intergroup conflict, the existing literature pays relatively little attention to the potential for change through the creation of spaces where opposing narratives and worldviews can be expressed and confronted within grassroots IC processes. In this context, only a limited number of studies have explored the possible effects of exposing individuals and groups to the narrative of the other side (Adwan, 2024; Ben David et al., 2017; Castro et al., 2023; Husnu et al., 2018; Kim & Lim, 2021; Kupermintz & Salomon, 2006; Ron & Maoz, 2013a; Zigenlaub & Sagy, 2020).

In light of this gap, the current review seeks to make a theoretical contribution by examining the interrelations between ethno-political conflicts, narratives, and IC. Among the studies surveyed, several point to the potential of confronting the narrative of the other within IC interventions as a means of fostering change (Bar-On, 2010; Ben David et al., 2017; Ellis et al., 2016; Hasler et al., 2023; Ron et al., 2020; Zigenlaub & Sagy, 2020). This potential is illustrated through the case study presented in the review, namely, the intractable conflict and communication processes between Israeli Jews and Palestinians, which demonstrates how IC can promote greater awareness of unequal power relations and social injustice. It also highlights the role of ‘dangerous stories’ that engage with the narratives of the suffering other in the context of the conflict (Bekerman & Zembylas, 2011; Ostovich, 2002, 2005; Ron & Maoz, 2013a; Zembylas & Bekerman, 2008). Together, these findings deepen our understanding of how IC processes can tackle manifestations of collective memories and traumas, expressions of the ethos and culture of conflict, and the master narratives of societies embroiled in intractable conflict. By addressing these elements, IC can help mitigate the harmful influence of narratives in sustaining ethno-political conflicts.

The current review may also offer an additional contribution to the field of intergroup communication. Research on IC has generally fallen into two main categories: quantitative studies that examine the impact of IC on participants’ attitudes toward the out-group (Burrows et al., 2022; Rosen, 2009; Salomon, 2004; Tropp et al., 2022; Van Assche et al., 2023), and studies that analyze discourse styles and patterns of interaction within IC processes (Bekerman, 2009; Hammack, 2009, 2011; Maoz, 2011; Ron, 2022). However, relatively little attention has been paid to the ideological ramifications of the IC experience, or to the ways in which participation in IC might shape perceptions of the nature of intergroup conflicts and possible avenues for their resolution (Alber, 2017; Ron et al., 2010).

In light of this gap, the current review seeks to illuminate the connections between participation in IC interventions, ideological positioning, and attitudes toward conflict resolution (See Table A1 in Appendix A). Section 3 of the review points to the potential of continuous involvement in IC processes to bring about a reconsideration of old ideological perspectives and attitudes regarding the other side, and to contribute to the creation of a new and different discourse between members of rival groups (Ron & Maoz, 2013a; Ron et al., 2010). The review also points to the ability of IC interventions to provide the participants with a deeper and more complex awareness of the conflict in which they are involved and of the proposed solutions to it-a shift that may be translated into new ideas and approaches to conflict resolution (Ellis, 2020; Ron & Maoz, 2013b; Thiessen & Darweish, 2018).

In terms of research focus, the contribution of the current review lies not only in its examination of key issues within the context of IC, such as group narratives and master-narratives, collective memory, ideology, and attitudes toward conflict resolution, but also in its exploration of the interconnections among these themes. By integrating insights from multiple fields, including narrative studies, IC, social and political psychology, conflict resolution, and peace research, this review offers a more holistic perspective. In doing so, it aims to deepen our understanding of both the potential and the limitations of bottom-up, grassroots processes of IC in complementing top-down political efforts at peacemaking and conflict resolution (Firchow & Dixon, 2025; Mitchell, 2009; Peshkopia, 2020) and in fostering reconciliation within ethno-political conflict settings.

### Limitations and Future Directions

In addition to the gaps in the existing literature mentioned above—specifically, the limited focus on the transformative potential of narratives through IC processes, the ideological ramifications of the IC experience, and how it may shape new perceptions concerning intergroup conflicts and their resolution—it is also important to note several limitations and gaps that characterize the current body of research. These include, but are not limited to: the scarcity of longitudinal studies, small sample sizes, specific limitations inherent to IC interventions and the platforms on which they take place, as well as gaps in the attention paid by narrative approaches to aspects such as identity and power asymmetries. Furthermore, while acknowledging the strengths of narrative literature reviews, such as the current one—including their flexibility, the emphasis placed on the author’s expertise, and their ability to integrate insights from various disciplines—their methodological limitations must also be acknowledged. These involve the potential for selection bias and interpretation bias resulting from the central place afforded to the author’s expertise, the absence of an explicit and predefined protocol, and consequently, the reduced transparency and limited reproducibility of such studies.

In order to achieve a more complete picture, there is clearly room for further research, including longitudinal studies tracking changes in narratives and perceptions over time, comparative studies, and various forms of experimental manipulation to examine the effects of exposure to out-group narratives. For example, the proposed longitudinal studies could incorporate follow-up tests and/or interviews in addition to standard pre-post designs. Such designs would allow for a more robust assessment of the durability of change over time and track the evolution of narrative complexity.

More specifically, as noted in Section 4 of the review, given the limited effectiveness of social media as a platform for complex IC processes, a potential research direction could address the capability of digital architectures and new technologies, such as VR and AI, to support these complex processes. Such studies could measure whether VR simulations or AI-driven perspective-taking can increase ideological openness by mitigating the perceived threat of the out-group narrative.

Lastly, while this review focuses on the Israeli–Palestinian conflict as a primary case study, its insights have broader, global implications. Future studies should explore the relationships between IC, narratives, ideology, and attitudes in additional conflict contexts. Such comparative research would allow for a more nuanced assessment of IC’s transformative potential, specifically by elucidating how contextual constraints shape the potential effects of contact experiences on participants’ ideology and their perception of reality within intergroup conflict settings.

## Figures and Tables

**Table 1:** Conflict narratives, IC models, and mechanisms of engagement.

Key Concepts	Coexistence Model	Joint Projects	Confrontational	Narrative Model
Narrative engagement			sh, ex, pr	sh, ex, pr, pt
Personal narrative	ia, st	soi		st
Ethos of conflict			sh, ex, pr	sh, ex, pr
Collective memory				wt
Master narrative			sh, ex, pr	sh, ex, pr

Mechanisms of engagement and change (abbreviations)—sh: sharing, ex: exposure, pr: processing, pt: perspective-taking, ds: ‘dangerous stories’, ia: interpersonal acquaintance, st: storytelling, soi: superordinate identity, wt: working through.

## Data Availability

No new data were created or analyzed in this study. Data sharing is not applicable to this article.

## Abbreviations

The following abbreviations are used in this manuscript:

IC	Intergroup communication
FTF	Face-to-face
CMC	Computer-mediated communicationR
VR	Virtual Reality
AI	Artificial Intelligence

## Appendix A

**Table A1:** Selected Examples of IC Studies.

Study (Author, Year)	IC Model	Setting	Population	Method	Key Conclusion
Al Ramiah and Hewstone (2013)	mixed, ch(ce)	Diverse	Diverse	Review	Potentially effective in pr, cp, cr
Dixon et al. (2005)	ch	Diverse	Diverse	Critique	Limitations of ch research
Maoz (2011)	ce, jp, con. nar	ig encounters	Diverse	Qualitative	Mapping onto four contact models
Pettigrew and Tropp (2006)	ch, mixed	Diverse	Diverse	ma	pr even under sub-optimal conditions
Saguy and Dovidio (2013)	mixed, con. ch(ce)	Diverse	Diverse, students	cs, le	ic content shaped by status differences
Tropp et al. (2022)	mixed, ch	Diverse	Youth	Review	Keys to successful youth ic

Abbreviations—ch: contact hypothesis, ce: coexistence, pr: prejudice reduction, cp: conflict prevention, cr: conflict resolution, ir: intergroup trlations, jp: joint ptojects, con: confrontational, nar: narrative, ig: intergroup, ma: meta-analysis, cs: correlational survey, le: laboratory experiment.

## References

[B1-behavsci-16-00231] Abu-Nimer M. (2004). Education for coexistence and Arab-Jewish encounters in Israel: Potential and challenges. Journal of Social Issues.

[B2-behavsci-16-00231] Adwan S., Carretero M., Perez-Manjarrez E. (2024). Dialogue between two conflicting historical narratives: Palestine, Israel, and the PRIME dual historical narratives project. Global perspectives on the role of dialogue in history education.

[B3-behavsci-16-00231] Ahmad M. N. (2025). Narrative literature reviews in scientific research: Pros and cons. Jordan Journal of Agricultural Sciences.

[B4-behavsci-16-00231] Alayan S., Riley C. (2024). The new Palestinian textbooks: A strategy for national identity and self-determination. Nations and Nationalism.

[B5-behavsci-16-00231] Alber J. (2017). Introduction: The ideological ramifications of narrative strategies. Storyworlds: A Journal of Narrative Studies.

[B6-behavsci-16-00231] Allport G. W. (1954). The nature of prejudice.

[B7-behavsci-16-00231] Al Ramiah A., Hewstone M. (2013). Intergroup contact as a tool for reducing, resolving, and preventing intergroup conflict: Evidence, limitations and potential. American Psychologist.

[B8-behavsci-16-00231] Amichai-Hamburger Y., Hasler B. S., Shani-Sherman T. (2015). Structured and unstructured intergroup contact in the digital age. Computers in Human Behavior.

[B9-behavsci-16-00231] Aouragh M. (2012). Social media, mediation and the Arab revolutions. TripleC: Communication, Capitalism & Critique.

[B10-behavsci-16-00231] Auchter J. (2018). Narrating trauma: Individuals, communities, storytelling. Millennium.

[B11-behavsci-16-00231] Bamberg M. (2011). Who am I? Narration and its contribution to self and identity. Theory and Psychology.

[B12-behavsci-16-00231] Bamberg M., De Fina A., Schiffrin D., Schwartz S., Luyckx K., Vignoles V. (2011). Discourse and identity construction. Handbook of identity, theory and research.

[B13-behavsci-16-00231] Bar-On D. (2006). Tell your life-story: Creating dialogue between Jews and Germans, Israelis and Palestinians.

[B14-behavsci-16-00231] Bar-On D. (2008). The others within us: Constructing Jewish-Israeli identity.

[B15-behavsci-16-00231] Bar-On D., Salomon G., Cairns E. (2010). Storytelling and multiple narratives in conflict situations: From the TRT group in the German-Jewish context to the dual-narrative approach of PRIME. Handbook on peace education.

[B16-behavsci-16-00231] Bar-On D., Adwan S., Rotberg R. I. (2006). The psychology of better dialogue between two separate but interdependent narratives. Israeli and Palestinian narratives of conflict: History’s double helix.

[B17-behavsci-16-00231] Bar-Tal D. (2013). Intractable conflicts: Socio-psychological foundations and dynamics.

[B18-behavsci-16-00231] Bar-Tal D., Mana A., Srour A. (2020). Conflict-supporting narratives and the struggle over them. Israeli and Palestinian collective narratives in conflict: A tribute to Shifra Sagy and her work.

[B19-behavsci-16-00231] Bar-Tal D., Chernyak-Hai L., Schori N., Gundar A. (2009). A sense of self-perceived collective victimhood in intractable conflicts. International Red Cross Review.

[B20-behavsci-16-00231] Bar-Tal D., Hameiri B. (2020). Interventions to change well-anchored attitudes in the context of intergroup conflict. Social and Personality Psychology Compass.

[B21-behavsci-16-00231] Bar-Tal D., Oren N., Nets-Zehngut R. (2014). Sociopsychological analysis of conflict-supporting narratives: A general framework. Journal of Peace Research.

[B22-behavsci-16-00231] Bar-Tal D., Salomon G., Rotberg R. I. (2006). Israeli-Jewish narratives of the Israeli-Palestinian conflict: Evolution, contents, functions, and consequences. Israeli and Palestinian narratives of conflict: History’s double helix.

[B23-behavsci-16-00231] Bekerman Z. (2009). Identity work in Palestinian-Jewish intergroup encounters: A cultural rhetorical analysis. Journal of Multicultural Discourse.

[B24-behavsci-16-00231] Bekerman Z., Maoz I. (2005). Troubles with identity: Obstacles to coexistence education in conflict ridden societies. Identity.

[B25-behavsci-16-00231] Bekerman Z., Zembylas M. (2009). Fearful symmetry: Palestinian and Jewish teachers confront contested narratives in integrated bilingual education. Teaching and Teacher Education.

[B26-behavsci-16-00231] Bekerman Z., Zembylas M. (2011). Teaching contested narratives: Identity, memory and reconciliation in peace education and beyond.

[B27-behavsci-16-00231] Ben David Y., Hameiri B., Benheim S., Leshem B., Sarid A., Sternberg M., Nadler A., Sagy S. (2017). Exploring ourselves within intergroup conflict: The role of intragroup dialogue in promoting acceptance of collective narratives and willingness toward reconciliation. Peace and Conflict: Journal of Peace Psychology.

[B28-behavsci-16-00231] Bennett W. L., Segerberg A. (2012). The logic of connective action: Digital media and the personalization of contentious politics. Information, Communication & Society.

[B29-behavsci-16-00231] Bhat R. M., Rajan P., Gamage L. (2023). Redressing Historical bias: Exploring the path to an accurate representation of the past. Social Science.

[B30-behavsci-16-00231] Bilali R. (2024). Narrative interventions in conflict settings: Harnessing the power of narratives to prevent violence and promote peace. European Review of Social Psychology.

[B31-behavsci-16-00231] Blagojević J., Šćekić R. (2022). The Arab spring a decade on: Information and communication technologies as a mass mobilization tool. Kybernetes.

[B32-behavsci-16-00231] Bliuc A.-M., Chidley A. (2022). From cooperation to conflict: The role of collective narratives in shaping group behaviour. Social and Personality Psychology Compass.

[B33-behavsci-16-00231] Bourhis J. (2017). Narrative literature review. The sage encyclopedia of communication research methods.

[B34-behavsci-16-00231] Bruner J. (1990). Acts of meaning..

[B35-behavsci-16-00231] Bruner J. (2008). Culture and mind: Their fruitful incommensurability. Ethos.

[B36-behavsci-16-00231] Buheji M., Hamza J. (2024). Branding resilience: Shaping Gaza’s global identity through narrative, solidarity, and advocacy. International Journal of Advanced and Multidisciplinary Social Science.

[B37-behavsci-16-00231] Burrows B., Tropp L. R., Dehrone T. A., Čehajić-Clancy S. (2022). How intergroup contact shapes intergroup attitudes and construals of relations between ethnic groups: Evidence from Bosnia and Herzegovina. Peace and Conflict: Journal of Peace Psychology.

[B38-behavsci-16-00231] Busselle R., Bilandzic H. (2009). Measuring narrative engagement. Media Psychology.

[B39-behavsci-16-00231] Canetti D., Elad-Strenger J., Lavi I., Guy D., Bar-Tal D. (2017). Exposure to violence, ethos of conflict, and support for compromise: Surveys in Israel, East Jerusalem, West Bank, and Gaza. Journal of Conflict Resolution.

[B40-behavsci-16-00231] Castells M. (2013). Communication power.

[B41-behavsci-16-00231] Castro E. M., Medina C., Suárez-Orozco C. (2023). “Everyone has their story”: Intergroup dialogue’s potential to cultivate connection through the sharing of migration narratives. Psychology in the Schools.

[B42-behavsci-16-00231] Cole E. (2007). Teaching the violent past: History education and reconciliation.

[B43-behavsci-16-00231] Cortés-Ramos A., Torrecilla García J. A., Landa-Blanco M., Poleo Gutiérrez F. J., Castilla Mesa M. T. (2021). Activism and social media: Youth participation and communication. Sustainability.

[B44-behavsci-16-00231] Curran J. (2002). Media and power.

[B45-behavsci-16-00231] Çakal H., Halabi S., Cazan A.-M., Eller A. (2021). Intergroup contact and endorsement of social change motivations: The mediating role of intergroup trust, perspective-taking, and intergroup anxiety among three advantaged groups in Northern Cyprus, Romania, and Israel. Group Processes & Intergroup Relations.

[B46-behavsci-16-00231] Čehajić-Clancy S., Halperin E. (2024). Advancing research and practice of psychological intergroup interventions. Nature Reviews Psychology.

[B47-behavsci-16-00231] Dajani Daoudi M. S., Barakat Z. M. (2013). Israelis and Palestinians: Contested narratives. Israel Studies.

[B48-behavsci-16-00231] De Fina A., Georgakopoulou A. (2011). Narrative and identities. Analyzing narrative: Discourse and sociolinguistic perspectives.

[B49-behavsci-16-00231] De Fina A., Schiffrin D., Bamberg M. (2006). Discourse and identity.

[B50-behavsci-16-00231] Dixon J., Durrheim K., Tredoux C. (2005). Beyond the optimal contact strategy: A reality check for the contact hypothesis. American Psychologist.

[B51-behavsci-16-00231] Dixon J., Durrheim K., Tredoux C. (2007). Contact and attitudes towards the principle and practice of racial equality. Psychological Science.

[B52-behavsci-16-00231] Duranti A. (2006). Narrating the political self in a campaign for U.S. congress. Language in Society.

[B53-behavsci-16-00231] Ehrmann M., Millar G. (2021). The power of narratives in conflict and peace: The case of contemporary Iraq. Civil Wars.

[B54-behavsci-16-00231] Ellis D. G. (2020). Talking to the enemy: Difficult conversations and ethnopolitical conflict. Negotiation and Conflict Management Research.

[B55-behavsci-16-00231] Ellis D. G., Maoz I. (2007). Online argument between Israeli Jews and Palestinians. Human Communication Research.

[B56-behavsci-16-00231] Ellis D. G., Ron Y., Maoz I., Kellett P. M., Matyok T. G. (2016). Communicative contact and the transformation of ethnopolitical conflicts. Communication and conflict transformation through local, regional, and global engagements.

[B57-behavsci-16-00231] Figueiredo A., Martinovic B., Rees J., Licata L. (2017). Collective memories and present-day intergroup relations: Introduction to the special thematic section [Editorial]. Journal of Social and Political Psychology.

[B58-behavsci-16-00231] Firchow P., Dixon P. (2025). The politics of measurement in the age of localization: Comparing “Top-Down” versus “Bottom-Up” metrics of reconciliation. International Political Sociology.

[B59-behavsci-16-00231] Gamaghelyan P., Rumyantsev S. (2021). The road to the second Karabakh war: The role of ethno-centric narratives in the Nagorno-Karabakh conflict. Caucasus Survey.

[B60-behavsci-16-00231] Golynchik E. O. (2020). The ethos of intractable interethnic conflict: Research approaches and prospects. RUDN Journal of Psychology and Pedagogics.

[B61-behavsci-16-00231] Greenhalgh T., Thorne S., Malterud K. (2018). Time to challenge the spurious hierarchy of systematic over narrative reviews?. European Journal of Clinical Investigation.

[B62-behavsci-16-00231] Grever M., van der Vlies T. (2017). Why national narratives are perpetuated: A literature review on new insights from history textbook research. London Review of Education.

[B63-behavsci-16-00231] Halabi R., Sonnenschein N. (2004). The Jewish-Palestinian encounter in a time of crisis. Journal of Social Issues.

[B64-behavsci-16-00231] Halperin E. (2011). Emotional barriers to peace: Negative emotions and public opinion about the peace process in the middle east. Peace and Conflict: Journal of Peace Psychology.

[B65-behavsci-16-00231] Halperin E., Bar-Tal D. (2011). Socio-psychological barriers to peace making: An empirical examination within the Israeli Jewish Society. Journal of Peace Research.

[B66-behavsci-16-00231] Halperin E., Hameiri B., Littman R. (2024). Psychological intergroup interventions: Evidence-based approaches to improve intergroup relations.

[B67-behavsci-16-00231] Hameiri B., Moore-Berg S. L., Guillard C., Falk E. B., Bruneau E. (2024). Perceived victimhood shapes support for interpartisan political violence in the United States. Psychology of Violence.

[B68-behavsci-16-00231] Hameiri B., Nadler A. (2017). Looking backward to move forward: Effects of acknowledgment of victimhood on readiness to compromise for peace in the protracted Israeli–Palestinian conflict. Personality and Social Psychology Bulletin.

[B69-behavsci-16-00231] Hameiri B., Sharvit K., Bar-Tal D., Shahar E., Halperin E. (2017). Support for self-censorship among Israelis as a barrier to resolving the Israeli-Palestinian conflict. Political Psychology.

[B70-behavsci-16-00231] Hammack P. L. (2009). Exploring the reproduction of conflict through narrative: Israeli youth motivated to participate in a coexistence program. Peace and Conflict: Journal of Peace Psychology.

[B71-behavsci-16-00231] Hammack P. L. (2010). The cultural psychology of Palestinian youth: A narrative approach. Culture & Psychology.

[B72-behavsci-16-00231] Hammack P. L. (2011). Narrative and the politics of identity: The cultural psychology of Israeli and Palestinian youth.

[B73-behavsci-16-00231] Hammack P. L., Pilecki A. (2012). Narrative as a root metaphor for political psychology. Political Psychology.

[B74-behavsci-16-00231] Haraldsson K. G., McLean K. C. (2022). “My story is not my own”: A qualitative analysis of personal and collective continuity. Qualitative Psychology.

[B75-behavsci-16-00231] Harel T. O., Maoz I., Halperin E. (2020). A conflict within a conflict: Intragroup ideological polarization and intergroup intractable conflict. Current Opinion in Behavioral Sciences.

[B76-behavsci-16-00231] Harel T. O., Nir N., Vandermeulen D., Maoz I., Halperin E. (2024). A threat to cohesion: Intragroup affective polarization in the context of intractable intergroup conflict. Journal of Conflict Resolution.

[B77-behavsci-16-00231] Hasler B. S., Amichai-Hamburger Y., Amichai-Hamburger Y. (2013). Online intergroup contact. The social net: Understanding our online behavior.

[B78-behavsci-16-00231] Hasler B. S., Hirschberger G., Shani-Sherman T., Friedman D. A. (2014). Virtual peacemakers: Mimicry increases empathy in simulated contact with virtual outgroup members. Cyberpsychology, Behavior, and Social Networking.

[B79-behavsci-16-00231] Hasler B. S., Ron Y., Weiss P. L., Halperin E., Hameiri B., Littman R. (2023). Improving intergroup relations through interactive media. Psychological intergroup interventions: Evidence-based approaches to improve intergroup relations.

[B80-behavsci-16-00231] Hässler T., Uluğ Ö. M., Kappmeier M., Travaglino G. A. (2021). Intergroup contact and social change: An integrated contact-collective action model. Journal of Social Issues.

[B81-behavsci-16-00231] Hebel-Sela S., Hameiri B., Tropp L. R., Moore-Berg S. L., Saxe R., Halperin E., Bruneau E. (2025). Virtual contact improves intergroup relations between non-Muslim American and Muslim students from the middle east, north Africa and southeast Asia in a field quasi-experiment. Communications Psychology.

[B82-behavsci-16-00231] Hilman N. S. N., Hafina A., Ilfiandra I. (2023). Increasing self-esteem of high school students through narrative counseling. Journal Psikologi Pendidikan dan Konseling.

[B83-behavsci-16-00231] Hogg M. A., Abrams D., Brewer M. B. (2017). Social identity: The role of self in group processes and intergroup relations. Group Processes & Intergroup Relations.

[B84-behavsci-16-00231] Hoter E., Shonfeld M., Asmaa N. G. (2012). TEC center: Linking technology, education and cultural diversity. Journal of Educational Technology.

[B85-behavsci-16-00231] Hughes S. S. (2020). Unbounded territoriality: Territorial control, settler colonialism, and Israel/Palestine. Settler Colonial Studies.

[B86-behavsci-16-00231] Husnu S., Mertan B., Cicek O. (2018). Reducing Turkish Cypriot children’s prejudice toward Greek Cypriots: Vicarious and extended intergroup contact through storytelling. Group Processes & Intergroup Relations.

[B87-behavsci-16-00231] Imperato C., Mancini T., Amichai-Hamburger Y. (2024). Solving online conflicts by sensing the other. The role of social presence and closeness in improving the effectiveness of online intergroup contact. International Journal of Intercultural Relations.

[B88-behavsci-16-00231] Imperato C., Schneider B. H., Caricati L., Amichai-Hamburger Y., Mancini T. (2021). Allport meets internet: A meta-analytical investigation of online intergroup contact and prejudice reduction. International Journal of Intercultural Relations.

[B89-behavsci-16-00231] Ivanović J., Stanković B., Žeželj I. (2024). When our history meets their history: Strategies young people in Serbia use to coordinate conflicting group narratives. European Journal of Social Psychology.

[B90-behavsci-16-00231] Jahan N., Naveed S., Zeshan M., Tahir M. A. (2016). How to conduct a systematic review: A narrative literature review. Cureus.

[B91-behavsci-16-00231] Jelić M., Čorkalo Biruški D., Ajduković D. (2021). Competing collective narratives in intergroup rapprochement: A transgenerational perspective. Journal of Social and Political Psychology.

[B92-behavsci-16-00231] Jeong H. Y., Uluğ Ö. M., Baysu G. (2023). The role of personal centrality of ingroup victimhood in intergroup relations and political agenda in Northern Ireland. Political Psychology.

[B93-behavsci-16-00231] Jirek S. L. (2016). Narrative reconstruction and post-traumatic growth among trauma survivors: The importance of narrative in social work research and practice. Qualitative Social Work.

[B94-behavsci-16-00231] Kabiljo L. (2019). Virtual reality fostering empathy: Meet the enemy. Studies in Art Education.

[B95-behavsci-16-00231] Kahanoff M. (2016). Jews and Arabs in Israel encountering their identities: Transformations in dialogue.

[B96-behavsci-16-00231] Kawai T., Fahlbusch J., Dienel H. L., Renn O., Renn R. (2022). Narratives for personal and collective transformations. Innovation: The European Journal of Social Science Research.

[B97-behavsci-16-00231] Kearney R. (2007). Narrating pain: The power of catharsis. Paragraph.

[B98-behavsci-16-00231] Kende J., Phalet K., Van den Noortgate W., Kara A., Fischer R. (2017). Equality revisited: A cultural meta-analysis of intergroup contact and prejudice. Social Psychological and Personality Science.

[B99-behavsci-16-00231] Khalaf A., Bekerman Z. (2024). Negotiating peace education: The dynamics of bottom-up/top-down integrated/bilingual education initiatives in Israel. Journal of Peace Education.

[B100-behavsci-16-00231] Kim N., Lim C. (2021). Meeting of minds: Narratives as a tool to reduce prejudice toward stigmatized group members. Group Processes & Intergroup Relations.

[B101-behavsci-16-00231] Klar Y., Baram H. (2014). In De*FENCE* of the in-group historical narrative in an intractable intergroup conflict: An individual-difference perspective. Political Psychology.

[B102-behavsci-16-00231] Kriesberg L. (2009). Changing conflict asymmetries constructively. *Dynamics of Asymmetric Conflict:* Pathways Toward Terrorism and Genocide.

[B103-behavsci-16-00231] Kupermintz H., Salomon G. (2006). Lessons to be learned from tesearch on peace education in the context of intractable conflict. Theory Into Practice.

[B104-behavsci-16-00231] Kvernbekk T., Bøe-Hansen O., Olmos P. (2017). How to win wars: The role of the war narrative. Narration as argument.

[B105-behavsci-16-00231] Leshem O. A., Halperin E., Siniver A. (2022). Societal beliefs, collective emotions, and the Palestinian-Israeli Conflict. Routledge companion to the Israeli-Palestinian conflict.

[B106-behavsci-16-00231] Li M., Leidner B., Hirschberger G., Park J. (2022). From threat to challenge: Understanding the impact of historical collective trauma on contemporary intergroup conflict. Perspectives on Psychological Science.

[B107-behavsci-16-00231] Liu J. H., Hilton D. J. (2005). How the past weighs on the presence: Social representations of history and their role in identity politics. British Journal of Social Psychology.

[B108-behavsci-16-00231] Lorenz-Spreen P., Oswald L., Lewandowsky S., Herwig R. (2023). A systematic review of worldwide causal and correlational evidence on digital media and democracy. Nature Human Behaviour.

[B109-behavsci-16-00231] Mana A., Srour A. (2020). Israeli and Palestinian collective narratives in conflict: A tribute to Shifra Sagy and her work.

[B110-behavsci-16-00231] Manfredi A., Puzella G., David L., Iolanda I., Jacopo M., Gabbiadini A. (2025). AI-driven digital humans for E-contact: A pre-registered study on reducing intergroup bias with generative artificial intelligence. Acta Psychologica.

[B111-behavsci-16-00231] Maoz I. (2004). Coexistence is in the eye of the beholder: Evaluating intergroup encounter interventions between Jews and Arabs in Israel. Journal of Social Issues.

[B112-behavsci-16-00231] Maoz I., Salomon G., Cairns E. (2010). Educating for peace through planned encounters between jews and Arabs in Israel: A reappraisal of effectiveness. Handbook on peace education.

[B113-behavsci-16-00231] Maoz I. (2011). Does contact work in protracted asymmetrical conflict? Appraising 20 years of reconciliation-aimed encounters between Israeli Jews and Palestinians. Journal of Peace Research.

[B114-behavsci-16-00231] Maoz I., McCauley C. (2008). Threat, dehumanization and support for retaliatory-aggressive policies in asymmetric conflict. Journal of Conflict Resolution.

[B115-behavsci-16-00231] Maoz I., Ron Y., Sharvit K., Halperin E. (2016). The road to peace? The potential of structured encounters between Israeli Jews and Palestinians in promoting peace. The Israeli-Palestinian conflict: A social psychology perspective—Celebrating the legacy of Daniel bar-Tal.

[B116-behavsci-16-00231] McAdams D. P., John O. P., Robins R. W. (2021). Narrative identity and the life story. Handbook of personality: Theory and research.

[B117-behavsci-16-00231] McAdams D. P., Josselson P., Lieblich A. (2006). Identity and story: Creating self in narrative.

[B118-behavsci-16-00231] McKeown S., Dixon J. (2017). The “contact hypothesis”: Critical reflections and future directions. Social and Personality Psychology Compass.

[B119-behavsci-16-00231] McLamore Q., Adelman L., Leidner B. (2019). Challenges to traditional narratives of intractable conflict decrease ingroup glorification. Personality and Social Psychology Bulletin.

[B120-behavsci-16-00231] McLean K. C., Boggs S., Haraldsson K., Lowe A., Fordham C., Byers S., Syed M. (2020). Personal identity development in cultural context: The socialization of master narratives about the gendered life course. International Journal of Behavioral Development.

[B121-behavsci-16-00231] McLean K. C., Lilgendahl J. P., Fordham C., Alpert E., Marsden E., Szymanowski K., McAdams D. P. (2017). Identity development in cultural context: The role of deviating from master narratives. Journal of Personality.

[B122-behavsci-16-00231] Mitchell C. (2009). Persuading lions: Problems of transferring insights from track-two exercises undertaken in conditions of asymmetry. *Dynamics of Asymmetric Conflict:* Pathways Toward Terrorism and Genocide.

[B123-behavsci-16-00231] Mor I., Ron Y., Maoz I. (2016). “Likes” for peace: Can Facebook promote dialogue in the Israeli-Palestinian conflict?. Media and Communication.

[B124-behavsci-16-00231] Murrar S., Brauer M. (2018). Entertainment-education effectively reduces prejudice. Group Processes & Intergroup Relations.

[B125-behavsci-16-00231] Murrar S., Brauer M. (2019). Overcoming resistance to change: Using narratives to create more positive intergroup attitudes. Current Directions in Psychological Science.

[B126-behavsci-16-00231] Myers S. (2021). Jerome Bruner, meaning making and education for conflict resolution: Why how we think matters.

[B127-behavsci-16-00231] Nagar I., Hoter E., Hasler B. S. (2021). Intergroup attitudes and interpersonal relationships in online contact between groups in conflict. Journal of Global Information Technology Management.

[B128-behavsci-16-00231] Nicolson M., Korkut U. (2021). The making and the portrayal of Scottish distinctiveness: How does the narrative create its audience?. International Migration.

[B129-behavsci-16-00231] Nolte-Laird R. (2022). Peacebuilding online dialogue and enabling positive peace.

[B130-behavsci-16-00231] Obradović S., Bowe M. (2021). The nation in context: How intergroup relations shape the discursive construction of identity continuity and discontinuity. The British Journal of Social Psychology.

[B131-behavsci-16-00231] Oren N., Nets-Zehngut R., Bar-Tal D. (2015). Construction of the Israeli-Jewish conflict-supportive narrative and the struggle over its dominance. Political Psychology.

[B132-behavsci-16-00231] Ostovich S. T., Confino A., Fritzsche P. (2002). Epilogue: Dangerous memories. The work of memory: New directions in the study of German society and culture.

[B133-behavsci-16-00231] Ostovich S. T. (2005). Dangerous memories and reason in History. KronoScope.

[B134-behavsci-16-00231] Papacharissi Z. (2002). The virtual sphere: The Internet as a public sphere. New Media and Society.

[B135-behavsci-16-00231] Park S. W., Moon H. (2022). Assessing identity formation via narratives. Current Psychology: A Journal for Diverse Perspectives on Diverse Psychological Issues.

[B136-behavsci-16-00231] Peshkopia R., Carey H. F. (2020). A bottom-up view at peacebuilding: Pragmatism, public opinion, and the individual as unit of analysis in postconflict societies. Peacebuilding paradigms: The impact of theoretical diversity on implementing sustainable peace.

[B137-behavsci-16-00231] Pettigrew T. F., Tropp L. R. (2006). A meta-analytic test of intergroup contact theory. Journal of Personality and Social Psychology.

[B138-behavsci-16-00231] Pettigrew T. F., Tropp L. R. (2011). When groups meet: The dynamics of intergroup contact.

[B139-behavsci-16-00231] Reimer N. K., Sengupta N. K. (2023). Meta-analysis of the “ironic” effects of intergroup contact. Journal of Personality and Social Psychology.

[B140-behavsci-16-00231] Ron Y., Yuval A., Yitzhaki D. (2022). Jewish-Palestinian encounters in Israel as awareness generators: The Jewish state and majority-minority relations from the perspective of the Jewish participants (Hebrew). Ink Inscribed 0n Worn Parchment: Education in Israel and the Jewish-Arab conflict.

[B141-behavsci-16-00231] Ron Y., Maoz I. (2013a). Dangerous stories: Encountering narratives of the other in the Israeli-Palestinian conflict. Peace and Conflict: Journal of Peace Psychology.

[B142-behavsci-16-00231] Ron Y., Maoz I. (2013b). Peacemaking through dialogue? Effects of intergroup dialogue on perceptions regarding the resolution of the Israeli-Palestinian conflict. Dynamics of Asymmetric Conflict.

[B143-behavsci-16-00231] Ron Y., Maoz I., Bekerman Z. (2010). Dialogue and ideology: The effect of continuous involvement in Jewish-Arab dialogue encounters on the ideological perspectives of Israeli-Jews. International Journal of Intercultural Relations.

[B144-behavsci-16-00231] Ron Y., Solomon J., Halperin E., Saguy T. (2017). Willingness to engage in intergroup contact: A multi-level approach. Peace and Conflict: Journal of Peace Psychology.

[B145-behavsci-16-00231] Ron Y., Suleiman C., Maoz I. (2020). Women for peace: Promoting dialogue and peace through Facebook?. Social Media + Society.

[B146-behavsci-16-00231] Rosen Y. (2009). Transformation of central and peripheral beliefs. Journal of Transformative Education.

[B147-behavsci-16-00231] Ross M. H., Calhon C., Price P., Timmer A. (2002). The political psychology of competing narratives: September 11 and beyond. Understanding September 11.

[B148-behavsci-16-00231] Rotberg R. I., Rotberg R. I. (2006). Building legitimacy through narrative. Israeli and Palestinian narratives of conflict: History’s double helix.

[B149-behavsci-16-00231] Saguy T., Dovidio J. F. (2013). Insecure status relations shape preferences for the content of intergroup contact. Personality and Social Psychology Bulletin.

[B150-behavsci-16-00231] Salomon G. (2004). A narrative-based view of coexistence education. Journal of Social Issues.

[B151-behavsci-16-00231] Sapir A., Alimi A. (2023). The elephant is in the room: Diversity regimes, liminality and play in a dialog program for Palestinian-Arab* and Jewish students in an Israeli university. Organization.

[B152-behavsci-16-00231] Scham P., Pogrund B., Ghanem A. (2013). Introduction to shared narratives—A Palestinian-Israeli dialogue. Israel Studies.

[B153-behavsci-16-00231] Schwab A. K., Sagioglou C., Greitemeyer T. (2018). Getting connected: Intergroup contact on Facebook. The Journal of Social Psychology.

[B154-behavsci-16-00231] Segal A., Keduri Y., Katz Jameson J., Hannah M. F. (2022). Going hybrid: Using Facebook groups for people-to-people dialogue. Contemporary trends in conflict & communication.

[B155-behavsci-16-00231] Selvanathan H. P., Leidner B., Petrović N., Prelić N., Ivanek I., Krugel J., Bjekić J. (2019). Wedialog.Net: A quantitative field test of the effects of online intergroup dialogue in promoting justice- versus harmony-oriented outcomes in Bosnia and Serbia. Peace and Conflict: Journal of Peace Psychology.

[B156-behavsci-16-00231] Sheehi L., Sheehi S. (2016). Enactments of otherness and searching for a third space in the Palestine-Israel matrix. Psychoanalysis, Culture & Society.

[B157-behavsci-16-00231] Sherif M. (1966). In common predicament: Social psychology of intergroup conflict and cooperation.

[B158-behavsci-16-00231] Sherif M., Harvey O. J., White B. J., Hood W. R., Sherif F. W. (1961). Intergroup conflict and cooperation: The robber’s cave experiment.

[B159-behavsci-16-00231] Smith J. R., Haslam S. A. (2017). Social psychology: Revisiting the classic studies.

[B160-behavsci-16-00231] Stephan W. G. (2014). Intergroup anxiety: Theory, research, and practice. Personality and Social Psychology Review.

[B161-behavsci-16-00231] Sukalla F., Bilandzic H., Bolls P. D., Busselle R. W. (2016). Embodiment of narrative engagement: Connecting self-reported narrative engagement to psychophysiological measures. Journal of Media Psychology: Theories, Methods, and Applications.

[B162-behavsci-16-00231] Suleiman R. (2004). Planned encounters between Jewish and Palestinian Israelis: A social-psychological perspective. Journal of Social Issues.

[B163-behavsci-16-00231] Swaab R. I., Lount R. B., Chung S., Brett J. M. (2021). Setting the stage for negotiations: How superordinate goal dialogues promote trust and joint gain in negotiations between teams. Organizational Behavior and Human Decision Processes.

[B164-behavsci-16-00231] Tajfel H., Turner J. C., Austin W. G., Worchel S. (1979). An integrative theory of intergroup conflict. The social psychology of intergroup relations.

[B165-behavsci-16-00231] Tajfel H., Turner J. C., Worchel S., Austin W. G. (1986). The social identity theory of intergroup behaviour. Psychology of intergroup relations.

[B166-behavsci-16-00231] Thiessen C., Darweish M. (2018). Conflict resolution and asymmetric conflict: The contradictions of planned contact interventions in Israel and Palestine. International Journal of Intercultural Relations.

[B167-behavsci-16-00231] Tropp L. R., White F., Rucinski C. L., Tredoux C. (2022). Intergroup contact and prejudice reduction: Prospects and challenges in changing youth attitudes. Review of General Psychology.

[B168-behavsci-16-00231] Tyushka A. (2021). Weaponizing narrative: Russia contesting Europe’s liberal identity, power and hegemony. Journal of Contemporary European Studies.

[B169-behavsci-16-00231] Uluğ Ö. M., Şen E., Sandal Önal E., Sefa Uysal M., Acar Y. G. (2023). An overview of the Turkish-Kurdish conflict narratives and their effects on intergroup relations. The political psychology of Kurds in Turkey.

[B170-behavsci-16-00231] Uluğ Ö. M., Cohrs J. C. (2017). Examining the ethos of conflict by exploring lay people’s representations of the Kurdish conflict in Turkey. Conflict Management and Peace Science.

[B171-behavsci-16-00231] Uluğ Ö. M., Lickel B., Leidner B., Hirschberger G. (2020). How do conflict narratives shape conflict- and peace-related outcomes among majority group members? The role of competitive victimhood in intractable conflicts. Group Processes & Intergroup Relations.

[B172-behavsci-16-00231] Ushomirsky I., Leshem O. A., Halperin E. (2023). Ethos of conflict in the international arena: Power predicts expression of threat for security and hope for peace in speeches of leaders of nations in conflict. Political Psychology.

[B173-behavsci-16-00231] Vaccari C., Valeriani A. (2021). Outside the bubble: Social media and political participation in western democracies.

[B174-behavsci-16-00231] Van Assche J., Swart H., Schmid K., Dhont K., Al Ramiah A., Christ O., Kauff M., Rothmann S., Savelkoul M., Tausch N., Wölfer R., Zahreddine S., Saleem M., Hewstone M. (2023). Intergroup contact is reliably associated with reduced prejudice, even in the face of group threat and discrimination. American Psychologist.

[B175-behavsci-16-00231] Ventsel A., Hansson S., Madisson M.-L., Sazonov V. (2019). Discourse of fear in strategic narratives: The case of Russia’s Zapad war games. Media, War & Conflict.

[B176-behavsci-16-00231] Walther J. B., Hoter E., Ganayem A., Shonfeld M. (2015). Computer-mediated communication and the reduction of prejudice: A controlled longitudinal field experiment among Jews and Arabs in Israel. Computers in Human Behavior.

[B177-behavsci-16-00231] Whittle M. (2023). The narrative practices of hostile environments: The story of the nation-as-family and the story of security. Frontiers in Human Dynamics.

[B178-behavsci-16-00231] Wilson C., Dunn A. (2011). The Arab springdigital media in the Egyptian revolution: Descriptive analysis from the Tahrir data set. International Journal of Communication.

[B179-behavsci-16-00231] Witteborn S. (2007). The expression of Palestinian identity in narratives about personal experiences: Implications for the study of narrative, identity, and social interaction. Research on Language and Social Interaction.

[B180-behavsci-16-00231] Zartman I. W. (2019). Analyzing intractability. I William Zartman: A pioneer in conflict management and area studies. Essays on contention and governance.

[B181-behavsci-16-00231] Zembylas M., Bekerman Z. (2008). Education and the dangerous memories of historical trauma: Narratives of pain, narratives of hope. Curriculum Inquiry.

[B182-behavsci-16-00231] Zigenlaub E., Sagy S. (2020). Encountering the narrative of the “other”: Comparing two types of dialogue groups of Jews and Arabs in Israel. Peace and Conflict: Journal of Peace Psychology.

[B183-behavsci-16-00231] Zúñiga X. (2003). Bridging differences through dialogue. About Campus.

[B184-behavsci-16-00231] Zúñiga X., Lopez G. E., Ford K. A. (2014). Intergroup dialogue: Engaging difference, social identities and social justice.

